# A simple and rapid LC-MS/MS method for therapeutic drug monitoring of cetuximab: a GPCO-UNICANCER proof of concept study in head-and-neck cancer patients

**DOI:** 10.1038/s41598-017-02821-x

**Published:** 2017-06-02

**Authors:** François Becher, Joseph Ciccolini, Diane-Charlotte Imbs, Clémence Marin, Claire Fournel, Charlotte Dupuis, Nicolas Fakhry, Bertrand Pourroy, Aurélie Ghettas, Alain Pruvost, Christophe Junot, Florence Duffaud, Bruno Lacarelle, Sebastien Salas

**Affiliations:** 1 Service de Pharmacologie et d’Immunoanalyse (SPI), CEA, INRA, Université Paris-Saclay, 91191 Gif sur Yvette, France; 2 Groupe de Pharmacologie Clinique & Oncologique (GPCO)-Unicancer, 101 rue de Tolbiac, 75013 Paris, France; 3 Clinical Pharmacokinetics Laboratory, SMARTc unit, Inserm S_911 CRO2, Aix Marseille Univ and La Timone University Hospital of Marseille, Marseille, France; 4 Medical Oncology Unit, La Timone University Hospital of Marseille, Marseille, France; 5 Department of Head & Neck Surgery, La Conception University Hospital of Marseille, Marseille, France; 6 Onco-Pharma, La Timone University Hospital of Marseille, Marseille, France

## Abstract

Administration of first-in-class anti-EGFR monoclonal antibody cetuximab is contingent upon extensive pharmacogenomic testing. However in addition to tumor genomics, drug exposure levels could play a critical, yet largely underestimated role, because several reports have demonstrated that cetuximab pharmacokinetic parameters, in particular clearance values, were associated with survival in patients. Here, we have developed an original bioanalytical method based upon the use of LC-MS/MS technology and a simplified sample preparation procedure to assay cetuximab in plasma samples from patients, thus meeting the requirements of standard Therapeutic Drug Monitoring in routine clinical practice. When tested prospectively in a pilot study in 25 head-and-neck cancer patients, this method showed that patients with clinical benefit had cetixumab residual concentrations higher than non-responding patients (i.e., 49 ± 16.3 µg/ml *VS*. 25.8 ± 17 µg/ml, p < 0.01 t test). Further ROC analysis showed that 33.8 µg/ml was the Cmin threshold predictive of response with an acceptable sensitivity (87%) and specificity (78%). Mass spectrometry-based therapeutic drug monitoring of cetuximab in head-and-neck cancer patients could therefore help to rapidly predict cetuximab efficacy and to adapt dosing if required.

## Introduction

Cetuximab has been approved for the treatment of several solid tumors such as metastatic colorectal cancer and squamous cell head and neck cancer, in association with chemotherapy^1, 2^. This IgG1 chimeric monoclonal antibody blocks extra-cellular EGFR1 receptor, thus interfering with downstream signaling pathways usually leading to differentiation, proliferation, angiogenesis and metastatic spreading processes. Of note, it has been demonstrated that RAS mutational status was a predictive marker of non-response. Indeed, tumor mutations on KRas and NRas will prevent signaling pathway to be interrupted, despite the upstream blockade of the EGFR1 receptor by cetuximab. As such, pharmacogenomic testing has been rendered mandatory because only patients with Ras wild-type cancer are eligible for cetuximab therapy. Despite the widespread use of such upfront biomarker-based strategy for selecting patients, about half of them will still fail to respond to cetuximab, either because other molecular determinants for response are yet to be discovered, or possibly because of insufficient drug exposure levels. Indeed, since the first registration phase I/II study published in 2007, dose/exposure/effects relationships have been evidenced with cetuximab, along with marked inter-patient variability in pharmacokinetics (PK) parameters^3^. More recently, several clinical reports have demonstrated how cetuximab PK parameters could influence treatment efficacy. In particular, the Paintaud group has repeatedly demonstrated how cetuximab clearance values could be predictive of survival (i.e., the lower the clearance, the longer the survival), both in head-and-neck^4^ and in colorectal cancer patients^5^. These clinical evidences all advocate for the implementation of therapeutic drug monitoring (TDM) strategies at bedside. Indeed, clearance is associated with drug exposure levels, making these latter a new potential marker to forecast cetuximab efficacy and to adapt dosing, in case of insufficient drug exposure in patients. Reaching this goal is contingent upon the availability of bioanalytical methods simple and rapid enough to meet the requirements of routine clinical use. Liquid chromatography - tandem mass spectrometry (LC-MS/MS) provides highly specific and precise bioanalytical tools for therapeutic drug monitoring of small molecules^6^. Application of LC-MS/MS to the quantification of therapeutic proteins is far more challenging due to the extreme diversity and complexity of endogenous plasma proteins^7^. In this context, sample preparation prior to LC-MS/MS is essential to avoid potential interferences. Methods based on affinity extraction by the antigen are proposed for robust monitoring of monoclonal antibodies in clinical studies, but require production of a costly stable-isotope-labeled version of the antibody to overcome the impact of variable extraction recovery^8, 9^. Regarding cetuximab, only one LC-MS/MS assay with prior immunoaffinity enrichment was reported in a previous study by our group at CEA^10, 11^. Methods for therapeutic drug monitoring must combine high throughput, high precision, simplicity and robustness at low cost for large scale clinical sample analyses. To this end, we have developed a bioanalytical LC-MS/MS assay of plasma cetuximab with simple sample preparation workflow, meeting the requirements of routine use in terms of cost- and time-effectiveness, and analytical performance. In this paper, we make a thorough presentation of this original technique and provide data from a prospective pilot-study designed to determine whether simple and rapid monitoring of residual and/or maximal plasma concentrations of cetuximab could be a relevant strategy to predict treatment efficacy in head-and-neck cancer patients.

## Results

### Analytical Procedure

#### Proteotypic peptides selection and LC-MS/MS detection

**Figure 1:**
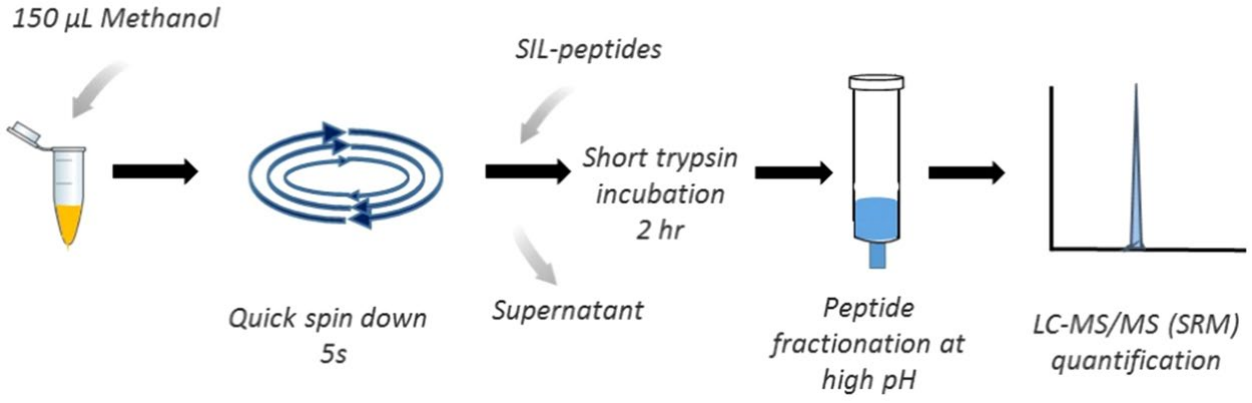
Simplified protocol for sample preparation prior to LC-MS/MS analysis. Figure 1 was prepared for the manuscript entitled “A simple and rapid LC-MS/MS method for therapeutic drug monitoring of cetuximab: proof of concept study in head-and-neck cancer patients”. All images were drawn by co-authors at CEA, France.

Identification and selection of best proteotypic peptides of cetuximab, used as surrogates of the protein target, was based on the recommendations for targeted proteomic experiments^12^, regarding sequence uniqueness and amino-acids composition. For absolute quantification of cetuximab during clinical applications, specificity was obtained by selecting peptides in the variable regions of the Fab fragments, and more precisely those containing the CDR sequence. Quantification relied on the two peptides LT3 and HT4 (Table 1), representative and specific of the light and the heavy chains respectively^10^. The two peptides were selected based on the signal intensity observed from a cetuximab digest analyzed in full scan MS mode (Q-TOF instrument). Peptides specificity for cetuximab was checked by a blast similarity search^10^. Stable-isotope-labeled version of the two selected proteotypic peptides (SIL-peptides) were used as internal standards (I.S.). SIL-peptides were spiked early in the analytical protocol, before the trypsin digestion (i.e. circumventing possible quantification bias due to any non-specific cleavages of HT4 and LT3 peptides during trypsin digestion) and the SPE fractionation steps (Fig. 1). A rapid UPLC separation of LT3 and HT4peptides was obtained within a 14 minutes total runtime. The mass spectrometer was operated in the SRM mode. Three SRM transitions were selected among the most intense observed from a cetuximab digest and eventually monitored for each peptide for high detection specificity. Product ions were preferentially selected with an m/z ratio higher than that of the parent ion (Table 1).

**Table 1:** MS parameters for SRM detection of HT4 and LT3 peptides as well as corresponding internal standards.

Peptide Sequence	Average mass (Da)	Precursor ion m/z	Product ion m/z	Collision energy (eV)	Retention time (min)
GLEWLGVIWSGGNTDYNTPFTSR	2570.8	857.6 (z = 3)	**908.6 (y16** ^+2^ **)**	16	8.8
			851.6 (y15^+2^)	16	8.8
			759.0 (y14^+2^)	16	8.8
GLEWLGVIWSGGNTDYNTPFTSR[^13^C_6_;^15^N_4_]	2580.8	860.9 (z = 3)	**913.6 (y16** ^+2^ **)**	16	8.8
			857.2 (y15^+2^)	16	8.8
			764.0 (y14^+2^)	16	8.8
ASQSIGTNIHWYQQR	1788.9	597.2 (z = 3)	**652.0 (y10** ^+2^ **)**	15	3.6
			708.6 (y11^+2^)	15	3.6
			752.2 (y12^+2^)	15	3.6
ASQSIGTNIHWYQQR[^13^C_6_;^15^N_4_]	1798.9	600.6 (z = 3)	**657.0 (y10** ^+2^ **)**	15	3.6
			713.6 (y11^+2^)	15	3.6
			757.2 (y12^+2^)	15	3.6

In bold: Product ion selected for quantification. Additional product ions are used for verification.

#### Sample preparation prior to LC-SRM analysis

A simple and fast workflow free of any affinity reagent was developed for routine patient monitoring of cetuximab concentration (Fig. 1). First step consists in an optimized pellet digestion protocol^13^. Methanol and acetonitrile solvent as well as perchloric acid were evaluated as the precipitant. Both solvent showed comparable recovery of cetuximab, and methanol was selected since it gave pellets more easily resuspended, prior to digestion, from the 50 µL plasma samples. Centrifugation time and speed were also assessed. Of note, reducing the centrifugation time to only a short 5 s spin was found decisive for robust recovery of cetuximab from the pellet (Fig. 1).

**Figure 2:**
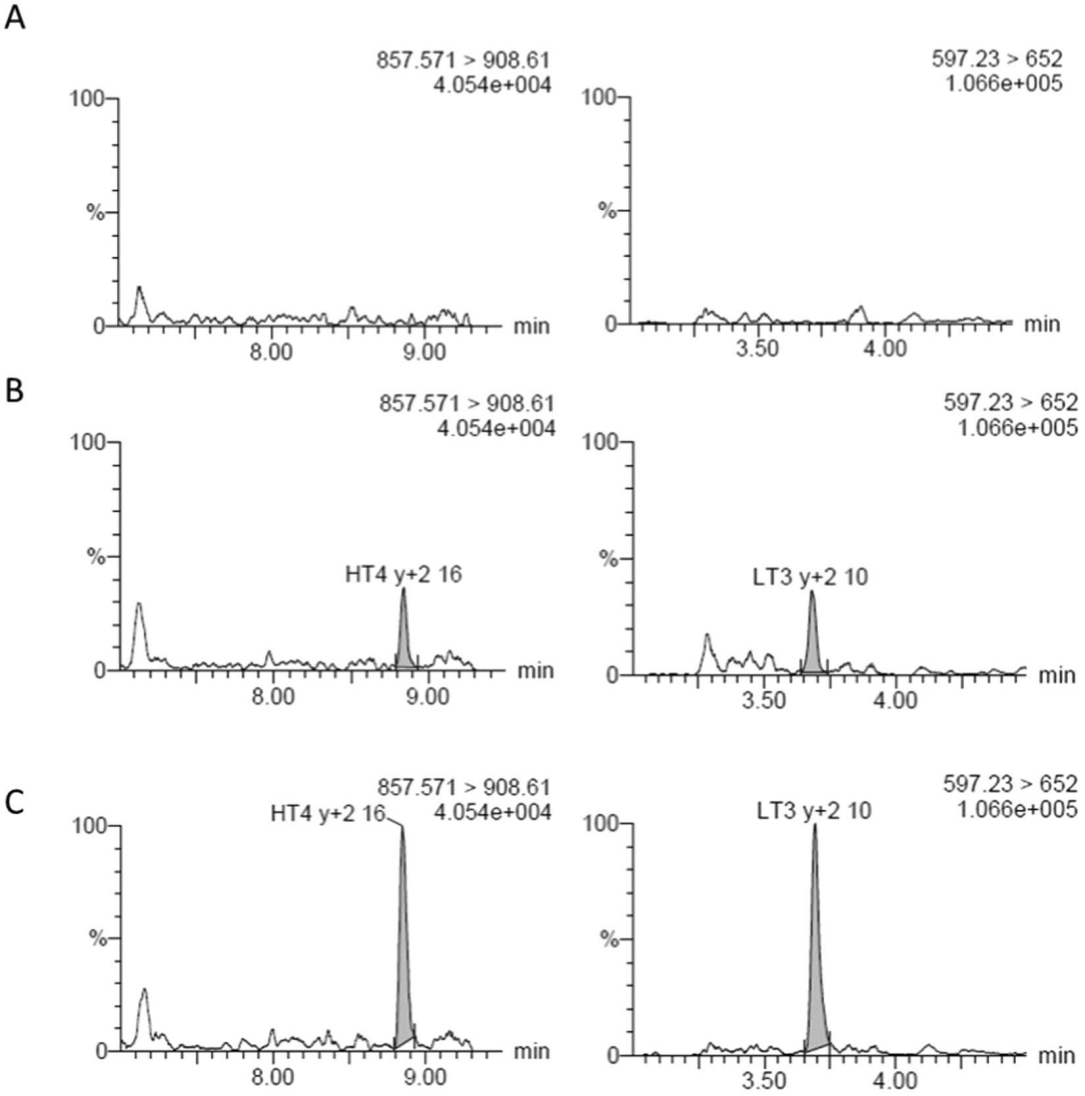
Chromatograms of HT4 (left) and LT3 (right) peptides illustrating signal in blank plasma samples (**A**), at LLOQ (**B**) and from a patient before cetuximab dosing (residual concentration) (**C**).

Protein digestion with trypsin is typically performed overnight, which is hardly compatible with routine TDM. Short digestion times were evaluated in combination with modified incubation conditions^14^. A protocol with incubation at 42 °C using 40 µg trypsin was selected as it resulted in robust signal for HT4 and LT3 after 2 hrs and similar intensity to overnight incubation. Reduction and alkylation steps were removed as no significant impact was observed on HT4 and LT3 signal and it improves the time to results. Under these conditions, no peptide degradation was observed for HT4 and LT3 with recoveries at 89.5% and 103.1%, respectively (Table 2). To further improve method robustness and sensitivity, complexity of the peptides mixture was reduced by solid phase extraction (SPE). Peptides fractionation on HLB cartridges operated at high pH was selected because it provides an orthogonal separation to the subsequent low-pH LC separation^15^ (Fig. 1). Washing and elution conditions were optimized for maximum recovery of HT4 and LT3 peptides. In the best conditions (i.e. washing and elution at 30% and 65% of methanol containing 2% NH_4_OH), SPE recovery of HT4 and LT3 peptides was found at 48.1% and 63.6%, respectively (Table 2). Next, ionization suppression was evaluated in human plasma. Ionization recovery of HT4 and LT3 was determined at 50.8% and 47.9% after comparison with signal in pure solutions, respectively (Table 2). Typical signals of HT4 and LT3 peptides in blank plasma samples, at lower limit of quantification (LLOQ) and in patient samples are illustrated in Fig. 2.

**Table 2:** Mean recoveries of LT3 and HT4 peptides in plasma samples.

	HT4		LT3	
	Mean	CV (%)	Mean	CV (%)
Digestion recovery	89.5	2.8	103.3	7.7
SPE extraction recovery	48.1	3.4	63.6	10.5
Ionization recovery	50.8	8.1	47.9	6.6
Total recovery	21.8	4.6	31.3	6.4

Mean recoveries were determined by measuring the peak area ratio of HT4 and LT3 peptides from a cetuximab digest spiked at 200 µg/mL, before and after incubation with trypsin, SPE extraction and confronted to peak area ratios from a pure solution. Means are from 3 experiments. CV: Coefficient of variation.

#### Method validation

Method validation was performed in human plasma according to current EMA guidelines for bioanalytical method validation^16^, including basic parameters of linearity, inter- and intra-assays precision and accuracy, LLOQ, matrix effect, selectivity and stabilities in human plasma.

**Figure 3:**
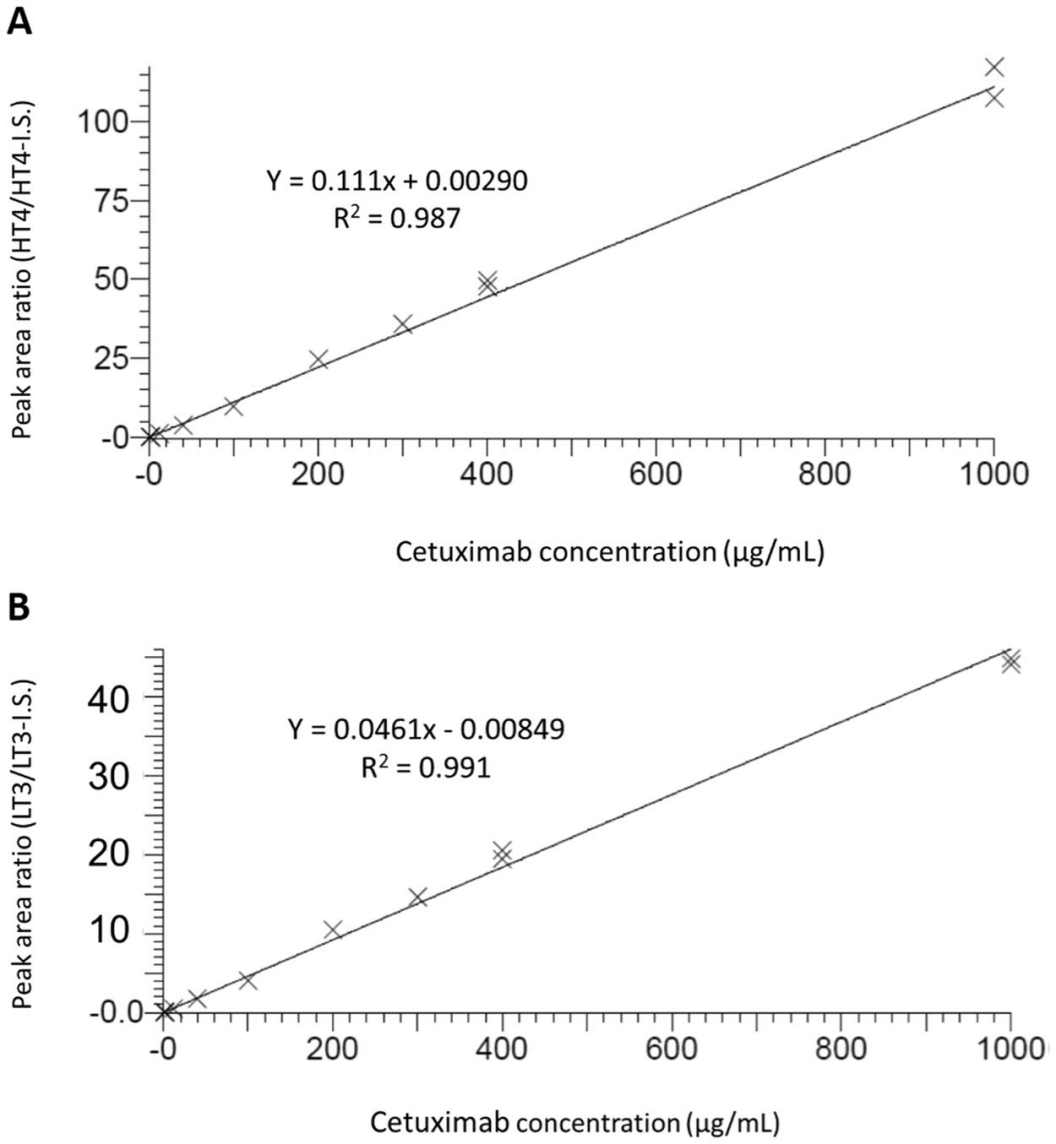
Linearity of Cetuximab quantification in human plasma. Results for HT4 (**A**) and LT3 (**B**) peptides are illustrated. Cetuximab was diluted in blank human plasma from 1 to 1000 µg/mL. Equation of the curve and correlation coefficient are indicated on the graphs.

Linearity of the protocol was assessed on each analytical run by spiking cetuximab in human plasma at 9 concentrations in the range of the calibration curve (Fig. 3). A linear regression model provided best fitting with mean regression coefficient at 0.9961 (+/−0.0018) over the 9 validation runs. Human plasma QCs samples spiked at LLOQ, LQC, MQC, and HQC of cetuximab were analyzed on three different days to determine precision and accuracy. Intra-day/inter-day precision and accuracy results at and above LLOQ are summarized in Tables 3 and 4. Matrix effects were assessed simultaneously to the intra-day experiment by repeated measurements in six individual batches of human plasma spiked with cetuximab at the LLOQ concentration. No impact of any of the six individual plasmas was observed, indicating the absence of significant variable matrix effect between different batches of plasma (Table 3). Selectivity was evaluated for both peptides in the 6 individual batches of blank human plasma. No signal interference was detected at the retention time of HT4 and LT3, for any of the three monitored SRM transitions. Stability of cetuximab was determined in spiked human plasma stored for 4 weeks at −80 °C, and at room temperature for 2 hours. No degradation was observed during these intervals. Accordingly, accuracy, precision and stability fulfilled to acceptance criteria, i.e., CV’s were <15% and accuracy in the 85–115% range.

**Table 3:** Intra-day and inter-day precision (CV%) and accuracy of cetuximab quantification.

QC value (µg/mL)	Intra-day			Inter-day		
	Mean	CV%	Accuracy	Mean	CV%	Accuracy
1	0.89*	8.0*	89.2*	0.933	14.7	93.3
1.8	1.66	9.5	92.0	1.74	9.7	96.8
90	79.7	2.8	88.6	88.4	9.5	98.3
175	178	2.5	101.7	180	12.4	103

Results from HT4 (A) and LT3 (B) peptides. *: Intra-day variability at LLOQ (1 µg/mL) was evaluated simultaneously with matrix effects in six individual batches of human plasma.

**Table 4:** Inter-day precision (CV%) and accuracy of cetuximab quantification.

QC value (µg/mL)	Intra-day			Inter-day		
	Mean	CV%	Accuracy	Mean	CV%	Accuracy
1	0.91*	6.5*	90.5*	0.88	6.4	88.4
1.8	1.54	6.4	85.5	1.72	14.3	95.8
90	79.3	4.6	88.1	91.6	11.1	102
175	184	5.1	105.2	188	5.9	107

Results from HT4 (A) and LT3 (B) peptides.

### Clinical Endpoints

Following CTCAE V3.0. criteria, 6 out of 25 patients (i.e., 24%) displayed or had displayed since the start of the treatment severe (i.e., grade-3) toxicities (anemia: 1 patient, thrombopenia: 1 patient, mucitis: 2 patients, renal toxicity: 2 patients). In addition, two patients exhibited severe radiodermatities. No grade-4 toxicities were observed here.

Following RECIST 1.0. assessment, 10 out of 25 patients (i.e., 40%) had progressive disease. The 15 remaining patients (60%) were lumped and categorized as patients with clinical benefit (stable disease: 5 patients (20%), partial response: 7 patients (28%), complete response: 3 patients (12%)).

Nine out of 25 patients died during the observation period. Among the deceased patients, average survival was 11 months from the start of cetuximab treatment.

### Therapeutic Drug Monitoring and exposure-pharmacodynamics relationships

**Figure 4:**
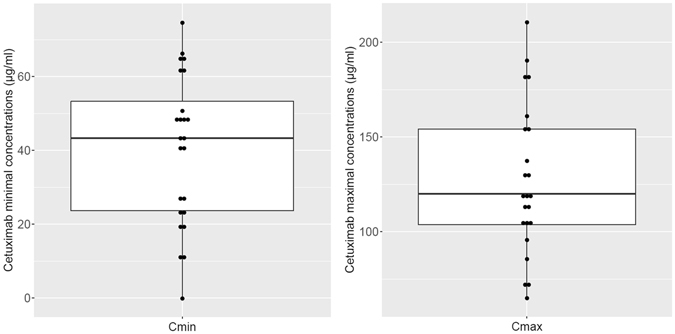
Dispersion of cetuximab concentrations after the end of the infusion (Cmax, right) and immediately before starting the next infusion (Cmin, left).

**Figure 5:**
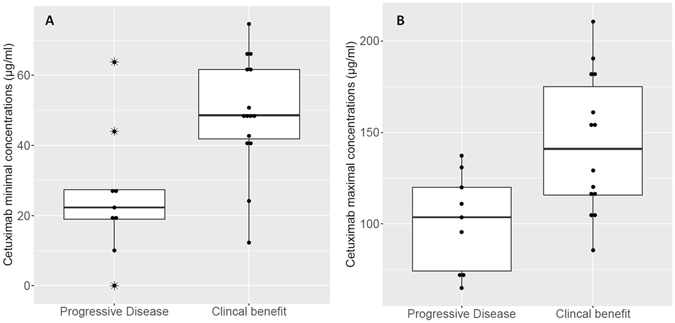
Boxplots comparing cetuximab exposure levels in patients depending on clinical outcome (i.e., Progressive Disease VS. Clinical Benefit). Figure 4-A shows residual concentrations and Fig. 4-B represents maximal concentrations.

**Figure 6:**
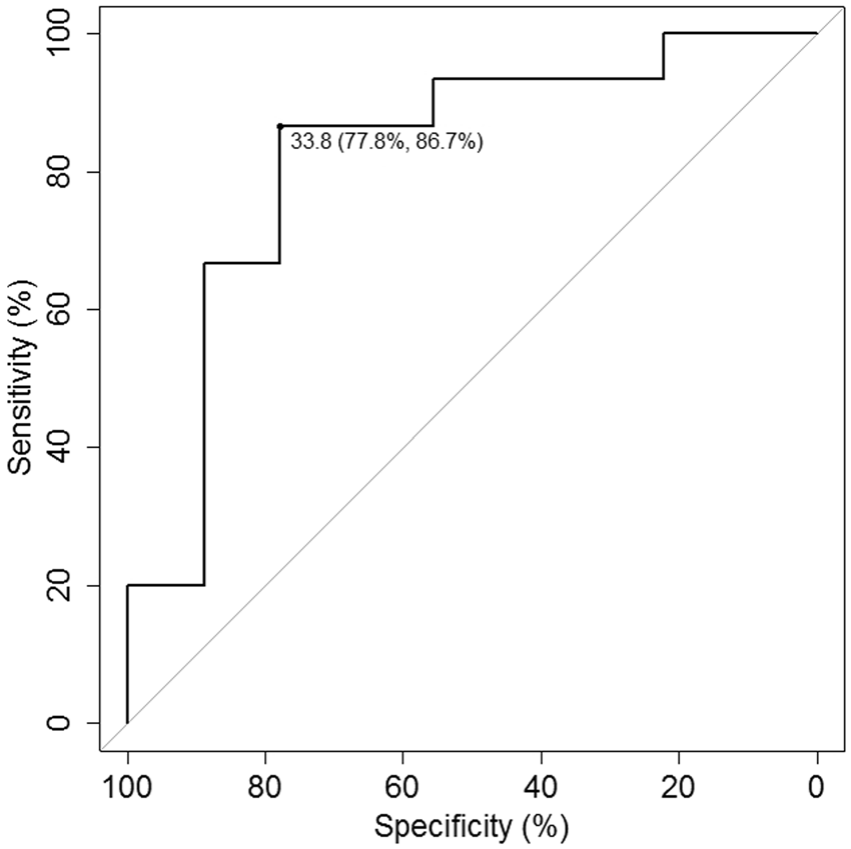
ROC curve analysis for cetuximab residual concentrations (Cmin) associated with a probability of clinical benefit.

At steady state, mean cetuximab residual concentrations were 40.3 ± 20.3 µg/ml (CV: 51%, range 0–74.6 µg/ml) and mean cetuximab maximal concentrations were 126.9 ± 39.6 µg/ml (CV: 31%, range 65.1–210.6 µg/ml) as shown in Fig. 4. When comparing exposure levels in patients with clinical benefit with that of non-responding patients (Fig. 5A and B), a statistical difference was found in both cetuximab residual concentrations (49.0 ± 16.3 µg/ml *VS*. 25.8 ± 17 µg/ml, p < 0.01, t test) and cetuximab maximal concentrations (143.7 ± 37.9 µg/ml *VS*. 100.9 ± 24.6 µg/ml, p < 0.05, t test). Further ROC analysis showed that 33.8 µg/ml was the Cmin threshold associated with a probability of clinical benefit with 78% of specificity and 87% of sensitivity (Fig. 6). Using this threshold, we found that 9 patients (i.e., 36%) were in the low Cmin group, whereas 16 patients (i.e., 64%) were in the high Cmin group. Regarding cetuximab concentrations at Tmax, ROC analysis on Cmax values defined a target Cmax of 113.2 µg/ml associated with clinical benefit, but with a specificity of 79% and a sensitivity of 67% only. In addition, Fischer Exact test confirmed that Cmin threshold of 33.8 µg/mL was significantly associated with clinical benefit (odds ratio 18.6; 95% confidence interval 1.9–327.8, p = 0.003,), but such association was not statistically confirmed for Cmax (odds ratio 6.6, 95% confidence interval 0.8–71.9, p = 0.07). No statistical difference was observed in cetuximab Cmin concentrations between patients with severe toxicities and patients with no toxicities (36.7 ± 21.2 µg/ml *VS*. 41.8 ± 20 µg/ml, p > 0.05, t test) nor in Cmax (117.1 ± 31.3 µg/ml *VS*. 130.3 ± 39.70 µg/ml, p > 0.05, t test).

## Discussion

Cetuximab is a paradigmatic drug for the development of a bioguided medicine in oncology, because it has been approved in patients with Ras wild-type tumors only^17^. Consequently, huge resources have been engaged to the widespread genotyping of Ras in all cancer patients scheduled for anti-EGFR therapy^18^. Although identifying mutated patients to be selected for other treatment modalities proved to be helpful, a significant subset of patients with wild-type cancer will still fail to respond to cetuximab-based regimen, despite favorable molecular and pharmacogenomic profile at the tumor level. A rising hypothesis is that beyond possible downstream deregulations at the tumor level, upstream pharmacokinetic issues, in particular low circulating drug levels, could explain poor efficacy in some patients treated with standard yet insufficient dosing^19^. To date, little is known about the pharmacokinetics of monoclonal antibodies^20^ and developing specific bioanalytical methods to measure plasma concentrations in routine patient has remained a challenging task for years^21^. Most of the methods currently used, either as part of registration clinical trials or academic studies are based upon immuno-assay such as canonical ELISA methods^22^, although other emerging immune-based techniques have been recently proposed^23^. Regarding cetuximab, all clinical reports published thus far were based upon ELISA measurement of drug levels in plasma using frequently in-house methods^3–5^. Although highly sensitive and fully validated, ELISA techniques suffer from a variety of downsides, ranging from time-consuming, multi-steps procedures to possible lack of robustness, cost-effectiveness issues unless large analytical batches are to be performed and possible operator-dependent or laboratory-dependent results if not performed in expert analytical centers. In addition, although commercial kits are now made available, only few clinical pharmacokinetics laboratories in cancer centers or in University-Hospitals have the expertise in properly handling in-house ELISA procedures, which can be a limitation to the widespread use of this technique as part of routine therapeutic drug monitoring at the bedside. In this respect, we have developed an original and simple LC-MS method to assay cetuximab in plasma which fulfills the contradictory criteria of sensitivity, simplicity, robustness and low cost for routine TDM. This original method is based on a fast sample preparation workflow coupled with a robust LC-MS/MS detection. The combination of a simple and rapid (i.e., 2 hrs) pellet digestion and a high-pH reverse phase peptides fractionation prior to the low-pH LC separation provided efficient quantification of residual and maximal cetuximab concentrations in all tested samples. The level of ionization suppression, determined at about 50% only, highlights the effectiveness of the fractionation protocol. It can be related to the complementarity between the high-pH fractionation and low-pH reverse phase chromatography, and the optimized washing and elution conditions during SPE. Quantification based on cetuximab calibration standards, processed similarly and simultaneously to the clinical samples, coupled to internal standardization by SIL-peptides spiked at an early step in the protocol demonstrated high repeatability and reproducibility, in line with the latest bioanalytical recommendations, and method’s performance in terms of analytical range and sensitivity was fully in line with available data regarding circulating cetuximab concentrations in patients with cancer^3–5^. Additionally, reproducibility, precision and transfer across laboratories of protein measurements with stable-isotope dilution and triple quadrupole instruments are already well demonstrated^24, 25^. The new protocol is based on a similar approach and could be thus easily transferred in any hospital laboratory equipped with standard triple quadrupole instruments. Of note, affinity reagents and full-length stable-isotope-labeled antibody^8, 9^ were avoided to facilitate inter-laboratory exchanges and/or maintain cost-effectiveness. In addition, all reagents are commercially available, including the cetuximab standard which was the pharmaceutical product (Erbitux®)obtained from the hospital pharmacy after dilution in human plasma. Several advantages are associated with the direct use of the pharmaceutical product as a standard: the drug is easily available at low cost from institution’s pharmacies (in this study, spare residual stock solution to prepare infusions were used instead of being discarded) and as a pharmaceutical form the solution is already highly characterized and standardized worldwide. These characteristics would ensure similar performance of the assay among different institutions, thus facilitating its transferability and ensuring a high potential for better harmonization of results between laboratories. Together with the good precision and accuracy of our assay, it paves the way for easier cross-validation of cetuximab assay among clinical pharmacokinetics laboratories. When tested as part of a pilot-study in cetuximab-treated patients, this original method proved its robustness and its full ability to discriminate non-responding patients from patients with clinical benefit, based upon the monitoring of residual and maximal concentrations, with no need to perform neither population-based identification of individual PK parameters nor simulation of complete exposure levels such as AUC determination. This method was used in actual routine setting of our institute, so as to test its performances in real-life conditions. For instance, sampling was performed by routine-care maids, and samples were next proceeded to the laboratory as routine tubes, with no absolute control on shelf and transport conditions from bedside to bench. Still, matrix effect was never an issue by our robust analytical conditions, despite the fact that samples may have stayed unattended before being transferred to the laboratory and that routine patients are much more polymedicated than usually observed in actual clinical trials. Indeed, no inclusion criteria but being treated with cetuximab was retained, and a variety of staging, co-medications, and exact localization of head-and-neck cancer were considered. Still, this diversity was not a confounding factor in this pilot-study. For instance, patients with metastatic diseases were found to be equally distributed in non-responding patients and in patients with clinical benefit (data not shown). Similarly, concomitant radiotherapy, or the use of carboplatin *VS*. cisplatin to be associated with 5-FU did not impact on clinical outcome either (data not shown). We found that cetuximab exposure levels were highly variable among patients. For instance, trough levels spanned over the 0–74.6 µg/ml range, whereas Cmax levels were in the 65.1–201.6 µg/ml range, meaning that for some patients, the residual concentrations in cetuximab were higher than the maximal concentrations reached at the end of the infusion for other patients. Conversely for some other patients, the maximal concentration was lower than the residual concentrations observed elsewhere. This discrepancy accounts for all the possible causes of variability in the PK of monoclonal antibodies, i.e. antigenic burden, changes in proteolytic degradation rates, influence of clinical covariates such as albumin or gender, presence of antibodies directed towards chimeric cetuximab, or even genetic polymorphisms (i.e., affecting the FcγR family), although these latter have been shown to have limited impact on cetuximab PK or clinical outcome^26, 27^. In addition, as already shown by other groups^4, 5^, we observed that efficacy was related to cetuximab pharmacokinetics because both Cmin and to a lesser extent Cmax levels were higher in stable/responding patients than in progressive disease patients. A marked difference in Cmin values was first evidenced (p < 0.01) and ROC analysis identified that residual concentrations above 33.8 µg/ml were significantly associated with clinical benefit, with 87% sensitivity and 78% specificity. Of note, a weaker statistical difference was observed as well between stable/responding and non-responding patients in their respective Cmax values. Still, further ROC analysis identified a target Cmax (113.2 µg/ml) to be reached to ensure clinical benefit with cetuximab, but with a sensitivity weaker than for Cmin (i.e., 67%). In addition, Fischer exact test was not significant for Cmax values, whereas it was significant for Cmin, thus suggesting that residual concentrations (trough levels)could be a more relevant threshold to be respected to achieve clinical benefit eventually. Of note, the fact that Cmin could be more critical than Cmax values for therapeutic efficacy with cetuximab is consistent with the PK/PD relationships of most monoclonal antibodies used to treat solid tumors. Massive antibodies hardly penetrate solid tumors indeed and therefore it is usually acknowledged that only extracellular targets of the external cell layer of the tumor mass will be easily reachable throughout time. In particular with cetuximab, it has been already shown in experimental models that distribution decreases with the increasing distance from blood vessels, and that little antibody is found in hypoxic regions such as tumor core^28^. Consequently, rather than reaching elevated Cmax values and therefore large quantities of plasma cetuximab that will be unable to penetrate in-depth tumor tissues eventually, ensuring a constant inhibition of the available EGFR targets on tumor surface with sustained trough levels is probably a better way to maximize efficacy. Here, all the patients displayed wild-type status on KRas and NRas and therefore should theoretically respond to anti-EGFR therapy. However, 40% of them had progressive disease, despite favorable downstream pharmacogenomic profile. Our data suggest therefore that patients with cetuximab residual concentrations below 34 µg/ml should probably have their dosing personalized to upper doses, so as to increase exposure levels and improve efficacy. Of note, here no such PK/PD relationship was evidenced with toxic-events. However, in this pilot-study most of the adverse-events we observed (i.e., hematological toxicity or renal impairment) could hardly be attributed to anti-EGFR therapy, but rather to the associated chemotherapy, i.e. cisplatin, carboplatin and 5-FU, despite efforts to customize dosing^27–29^. In this respect, the fact that no relationship could be evidenced here between cetuximab exposure levels and treatment-related side effects cannot lead to the conclusion that such link does not exist, owing to the limited number of patients included in this preliminary study and the fact that here, no typical cetuximab-related toxicities (e.g., hypomagnesaemia, skin toxicity) were recorded to fully test this hypothesis. Finally, the number of patients enrolled in this pilot-study and the duration of the observation period were both too small to evaluate properly survival. However, we observed that among the 9 deceased patients, dead individuals in the low Cmin subgroup (4 patients) had a mean survival of 4.8 months (range: 4–15 months), whereas the ones in the high Cmin subgroup (5 patients) had a mean survival of 13.1 months (range: 10–42 months). Although to be considered only as a trend with no statistical meaning, this observation was in line with the positive impact we evidenced between higher residual concentrations of cetuximab and clinical efficacy.

## Conclusion

Although monocentric and conducted on a limited subset of patients, this prospective proof-of-concept study strongly suggests that monitoring cetuximab residual concentrations using an original mass spectrometry technique is feasible, rapid and cheap, while providing relevant information to forecast clinical efficacy, in addition to standard downstream pharmacogenomic testing at the tumor level. This method could therefore be used as a convenient tool for implementing therapeutic drug monitoring strategy with cetuximab for early decision-making (i.e., maintaining standard dosing or increasing cetuximab dosing beyond 250 mg/m² QW) so as to ensure a maximal efficacy in patients with head-and-neck cancer.

## Material and Methods

### Analytical Procedure

#### Materials

Chemicals and Reagents. Trypsin from bovine pancreas TPCK Treated (reference T1426), analytical grade formic acid (FA), ammonium hydroxide, Ammonium bicarbonate, HPLC-grade acetonitrile (ACN) were from Sigma-Aldrich (Sigma Chemical Co., St Louis, MO, USA). SIL-peptides were from ThermoFisher Scientific (Paisley, UK). All experiments were done in LoBind tubes from Eppendorf (Hamburg, Germany). Cetuximab (Erbitux®, Merck KGaA) was obtained from the La Timone University Hospital’s pharmacy, provided as a 5 mg/mL solution for infusion. Calibration standard and QCs were obtained by diluting the 5 mg/mL solution in human plasma from healthy volunteers (Etablissement Français du Sang, Marseille).

#### Analytical Workflow

Pellet digestion was optimized from the procedure previously described^13^. A volume of 50 µl of human plasma samples was vortex-mixed with 150 µl of methanol, followed by quick spin down (5 s) on a mini centrifuge. The supernatant was decanted carefully, and the protein pellets were suspended with 150 µl of 50 mM ammonium bicarbonate in water for 10 minutes. Then, 10 µL of SIL-peptides at 1.25 µM and 20 µl of trypsin at 2 mg/mL were added (i.e., corresponding to a protein-to-trypsin ratio of 1/75). Samples were incubated at 42 °C for 2 hours. Tryptic digests were mixed with 300 µl of 2% ammonium hydroxide before SPE on HLB1cc/3 mg. Washing of SPE cartridge was performed with 1 mL of water/methanol (70/30) containing 2% Ammonium hydroxide, before elution with 0.5 mL water/methanol (35/65) containing 2% Ammonium hydroxide. Following evaporation under a stream of gaseous nitrogen, the dry residue was re-dissolved in 50 µL Water/acetonitrile (95/5) containing 0.1% formic acid, centrifuged for 5 min at 20000 g and transferred into an LC vial.

#### LC-MS/MS analysis

LC-MS/MS analyses were performed on a XEVO TQ-S triple quadrupole mass spectrometer (Waters, USA) controlled by Masslynx software (version 4.1). The instrument was linked to an Acquity I-Class LC system (Waters, USA). Chromatography was performed using a gradient combining solvent A (0.1% formic acid in water) and solvent B (0.1% formic acid in acetonitrile). Peptides were separated on an Acquity UPLC BEH Shield C18 column, 2.1 mm × 50 mm, 1.7 μm, (Waters, USA). Peptide separation was achieved using a gradient from 5 to 33% B over 10 min, at a flow rate of 500 μl/min and a temperature of 50 °C. MS data were acquired in positive mode with an ion spray voltage of 3000 V, a source and a desolvation temperature set to 150 °C and 225 °C, respectively. Cone voltage was set to 40 V and optimal collision energy (CE) was determined at 15 and 16 eV (Table 1). Scheduled SRM acquisitions were performed with Q1 and Q3 quadruples operating at unit resolution. The dwell time for HT4 and LT3 was set to 0.052 and 0.025 s, respectively corresponding to about 15 acquisition points per LC peak. MS data from measurements in patient samples have been deposited in the PeptideAtlas SRM Experiment Library (PASSEL) (Identifier PASS00983)^30^. Peak area measurements of cetuximab peptides from the calibration curve illustrated in Fig. 3 and from all patient samples are illustrated in Tables S1 and S2.

#### Quantification protocol

An external calibration curve was prepared in pooled human plasma spiked with cetuximab. To overcome the impact of partial recovery during trypsin digestion or peptide extraction, the calibration curve was processed similarly and simultaneously to the clinical samples. Moreover, SIL-peptides were spiked at the earliest in the analytical protocol, i.e. before trypsin digestion of the re-suspended protein pellet. SIL-peptides were also used to increase specificity of the peptide signal detection. For a given peptide, validation of the measurement requires that two specificity criteria are met: (i) the variation of the retention time should be within 0.1 min in replicate injections with strict co-elution with the SIL-peptide and similar peak shape; (ii) the relative ratio (i.e., a given transition by dividing its peak area by the peak area of another transition) of the analyte compared with the relative ratio of the SIL-peptides should not be significantly different (±25%). Quantification of cetuximab was done through both HT3 and LT4 peptides measurements. Cetuximab concentration was established as the mean of LT3 and HT4 measurements, after verification of a difference between HT3 and LT4 determination below 20%.

#### Method validation

The method was validated according to the EMA guideline on bioanalytical method validation^16^. Selectivity and matrix effect were evaluated by analyzing six different batches of human plasma. For each batch, two samples were analyzed: a blank sample and a sample spiked with cetuximab at the LLOQ and IS. No interfering components were considered when the signal was less than 20% of the LLOQ for the analyte. Matrix effect was assessed by the coefficient of variation and accuracy of the measured amounts, similarly to the intra-day/inter-day precision and accuracy study. Linearity was studied by analyzing calibration standards on nine separate days prepared by spiking cetuximab in human plasma at 9 concentrations between 1 and 1000 µg/mL. Calibration curves were established using peak area ratio HT4/IS or LT3/IS versus cetuximab concentations. Slopes, intercepts and regression coefficients (r²) were obtained by linear regression analysis. The best weighting factor was selected in order to minimize the sum of squared residuals. Back-calculated standard amounts could not differ from ±15% of the theoretical value (except ±20% for the lowest standard). The intra-day study was performed by assaying the four QC levels six times during the same analytical run, while the inter-day study was performed by assaying the four QC level six times on 3 separate days. Mean measured amounts and their SD were calculated. Precision was determined by the coefficient of variation (CV = (SD/mean) × 100), while accuracy was expressed as (measured concentration/theoretical concentration) × 100. Acceptance criteria were: accuracy from 85 to 115% of the nominal value (except 80–120% for the LLOQ) and CV lower than 15% (except 20% for the LLOQ). LLOQ was defined as the lowest quantity of analyte which can be determined with acceptable accuracy and precision (accuracy from 80 to 120% and CV lower than 20%). Stability of QCs stored at −80 °C for 4 weeks or 2 hours at room temperature was assessed in triplicate on the three QC levels (low, high) by measuring the accuracy.

### Patients

To test the TDM procedure in real-life settings, being an adult treated with cetuximab for head-and-neck cancer and having signed an informed consent were the only inclusion criteria in this study. A total of 25 patients (22 M, 3 F, mean age: 69 years (range 48–85)) hospitalized in the Medical Oncology Unit of La Timone University Hospital of Marseille for head-and-neck epidermoïd cancer (mouth: 7, larynx: 4, oropharynx: 4, others: 10) all being treated with a cetuximab-containing regimen between December 2015 and June 2016 were enrolled in this pilot study. All patients underwent prior pharmacogenomic testing (i.e., KRas and NRas mutational status determination) to determine their eligibility to be treated with cetuximab. Nine patients had locally advanced cancer and 4 patients showed metastatic disease. Five patients had been previously treated with radiotherapy, nine patients have been treated previously with at least one line of cytotoxic chemotherapy. All patients had signed an informed consent prior to extra-sampling of two blood samples to measure cetuximab levels, and local ethic committee (*Comité de Protection des Personnes Sud-Méditerranée*) approval was obtained prior to start the study. All experiments were conducted following current guidelines. Blood sampling was performed following standard nurse practice in the Medical Oncology Unit by trained maids. Bioanalytical analysis for cetuximab was performed in an ISO15189-labelled laboratory. Collection of clinical endpoints (i.e., efficacy, toxicity) was performed following standard guidelines in oncology (i.e., RECIST 1.0 and CTCAE V3.0 guidelines, respectively) for patients with head-and-neck cancers.

### Treatment

Apart from the two PK blood sampling, patient care was performed according to standard guidelines and routine care of the institution. Cetuximab was therefore administered following standard recommendations in patients with Ras wild-type head-and-neck cancer, i.e. 250 mg/m² weekly, in association with a platinum derivative (cisplatin or carboplatin) plus 5-FU. In our institute, 5-FU dosing in head-and-neck cancer patients is customized according to the DPD status of the patients, following an upfront geometric adaptive dosing strategy already published^29^. Similarly, both cisplatin and carboplatin dosing are tailored in real-time using a Bayesian adaptive strategy with feedback following a methodology previously described^31, 32^. One patient received paclitaxel rather than the platinum derivative/5-Fu doublet with cetuximab. Seven patients received concomitant radiotherapy.

### PK sampling

Sampling was performed after at least 2 months of treatment, i.e. after that a minimum of 4 courses of cetuximab had been administrated, so as to ensure that steady-state has been reached. Two blood samples were withdrawn immediately on Li heparinate tubes after the administration of cetuximab (=Cmin, or trough levels) and immediately after the end of the infusion (=Cmax). Blood samples were transferred to the laboratory and immediately centrifuged (3000 g, 15 min 4 °C), plasma fraction was next isolated and kept at −80 °C until analyzed as described above.

### Clinical endpoints

Toxicity was monitored prospectively following Common Terminology Criteria for Adverse Events V3.0 (CTCAE) grading. Efficacy was evaluated following standard RECIST 1.0. criteria for head-and-neck cancer three months after introducing cetuximab. Because of the small sample size and to gain statistical power, treatment efficacy was categorized in only two groups: non-responding patients (i.e., progressive disease) and patients with clinical benefit (i.e., stable disease, partial response, complete response).

### Statistical Analysis

Differences in Cmin and Cmax values between patients with clinical benefit and patients with progressive disease were tested using standard t-test (Sigma Stat, Germany). Owing to the small number of patients (n = 25), a p value < 0.05 was regarded as statistically significant. Similarly, differences in exposure levels between patients with and patients without severe toxicities were investigated using t-test. Comparisons of proportions were performed using Fisher exact test considering the small number of patients. Finally, receiver operating characteristic (ROC) curve analysis was performed to assess a discrimination potential of trough and maximal cetuximab plasma levels for clinical benefit (R, R Development Core Team, Austria).

## Electronic supplementary material

Dataset1 TableA

Dataset1 TableB

Dateset2 Table A

Dateset2 Table A
